# Deficiency and Excess of Folic Acid Intake Promote Colorectal Carcinogenesis in AOM/DSS-Treated Mice: Roles in Uracil Misincorporation and DNA Methylation

**DOI:** 10.3390/nu18081187

**Published:** 2026-04-09

**Authors:** Qinghan Ren, Yunfei Ma, Zhenshu Li, Qi Wu, Tongtong Li, Xin He, Wen Li, Yongjie Chen, Fei Ma, Jing Yan, Guowei Huang

**Affiliations:** 1 Department of Nutrition and Food Science, School of Public Health, Tianjin Medical University, Tianjin 300070, China; renqinghan0223@tmu.edu.cn (Q.R.); mayunfei@tmu.edu.cn (Y.M.); lizhenshu@tmu.edu.cn (Z.L.); wq17702280916@tmu.edu.cn (Q.W.); litongtong@tmu.edu.cn (T.L.); he.xin@tmu.edu.cn (X.H.); liwen828@tmu.edu.cn (W.L.); 2 Key Laboratory of Prevention and Control of Major Diseases in the Population, Ministry of Education, Tianjin Medical University, Tianjin 300070, China; 3 Tianjin Key Laboratory of Environment, Nutrition and Public Health, Tianjin 300070, China; 4 The Province and Ministry Co-Sponsored Collaborative Innovation Center for Medical Epigenetics, Tianjin 300070, China; 5 Department of Epidemiology & Biostatistics, School of Public Health, Tianjin Medical University, Tianjin 300070, China; chenyongjie@tmu.edu.cn (Y.C.); mafei@tmu.edu.cn (F.M.); 6 Department of Health Management, School of Public Health, Tianjin Medical University, Tianjin 300070, China

**Keywords:** folic acid, deficiency, excess, colorectal cancer, uracil misincorporation, DNA methylation, mouse

## Abstract

**Background/Objectives:** Colorectal cancer (CRC) is a leading cause of cancer-related mortality worldwide, yet the association between folic acid (FA) intake and CRC risk remains controversial. This study investigated the effects of varying dietary FA levels on colorectal carcinogenesis and the underlying mechanisms. **Methods:** BALB/c mice were fed diets containing FA at <0.1, 2.0, 6.0, 8.0, or 20.0 mg/kg for 14 weeks. After 4 weeks, colorectal tumorigenesis was induced using the azoxymethane/dextran sulfate sodium (AOM/DSS) protocol. Tumor multiplicity, maximum tumor diameter, tumor volume, colorectal length, histopathology, and cell proliferation were assessed. Mechanistic assessments included uracil misincorporation, thymidylate synthase (TS), telomere attrition, genome-wide DNA methylation, RAP1 signaling, immune-related markers, and inflammatory cytokines in colorectal tissues. **Results:** Both FA deficiency (<0.1 mg/kg) and excess (8.0/20.0 mg/kg) increased colorectal tumor burden, with increased tumor number, larger maximum diameter, greater tumor volume, shortened colorectal length, and enhanced cell proliferation, whereas the 6.0 mg/kg diet group showed the lowest tumor burden. FA deficiency reduced TS expression, elevated deoxyuridine monophosphate (dUMP) levels, decreased deoxythymidine monophosphate (dTMP) levels, increased uracil misincorporation, and exacerbated telomere attrition, as evidenced by shortened telomeres and increased damage. In contrast, excessive FA intake induced Rap1 GTPase-activating protein (*RAP1GAP*) hypermethylation, reduced Rap1GAP expression, enhanced RAP1 activity, and upregulated programmed death-ligand 1 (PD-L1) and cytotoxic T-lymphocyte-associated protein 4 (CTLA4) expression. **Conclusions:** Dietary FA can exhibit a U-shaped association with colorectal carcinogenesis, with protective effects observed within an optimal range. FA deficiency and excess may drive tumor development through distinct molecular pathways involving uracil misincorporation-induced telomere attrition and DNA methylation-mediated immunosuppression, respectively.

## 1. Introduction

Colorectal cancer (CRC) ranks as the third most common and second deadliest malignancy globally, representing a major and increasing public health burden [1]. The global incidence of CRC continues to rise, and due to the often insidious nature of early-stage symptoms, a majority of patients are diagnosed at intermediate or advanced stages, underscoring the critical importance of early prevention strategies [2]. Although genetic mutations are key drivers of CRC, non-genetic drivers, particularly dietary and lifestyle factors, are critically important [3]. Among nutritional factors, folate has attracted significant attention for its potential effects against CRC [4]. Folic acid (FA) is most widely recognized for its role in the biosynthesis of nucleotides (purines and thymidine), thereby promoting DNA synthesis and repair, as well as remethylating homocysteine to form methionine [5]. Since humans cannot synthesize folate endogenously, it must be obtained from dietary sources or supplements, primarily in the form of FA [4].

Research over the past several decades has yielded inconsistent findings regarding the relationship between FA intake and CRC risk. While early meta-analyses suggested a protective role against CRC [6], subsequent clinical evidence, such as the Aspirin/Folate Polyp Prevention Study, found that 1 mg/day of FA failed to reduce adenoma recurrence and potentially increased the risk of advanced lesions [7]. Mechanistically, FA deficiency may initiate tumorigenesis in healthy mucosa, yet supplementation often accelerates the progression of established pre-neoplastic lesions [8]. This paradox is further reflected in population-level data showing a rise in CRC incidence following mandatory FA fortification [9]. These conflicting findings underscore the complexity of FA’s role in CRC and highlight the need for more systematic investigation into its context-dependent effects.

At the molecular level, folate deficiency causes a reduction in thymidine synthesis and a subsequent imbalance in the deoxyribonucleotide pool, resulting in uracil misincorporation during DNA replication and repair [8]. Such misincorporation can induce double-strand breaks, deletions, chromosomal fragmentation, micronucleus formation, and loss of heterozygosity, potentially explaining the elevated cancer risk linked to folate deficiency [10]. Telomeres, the protective nucleoprotein complexes at chromosome ends, are susceptible to uracil misincorporation and repair defects due to their thymidine-rich sequences [11]. Damage to telomeres may in turn trigger genomic instability through gene amplification, large-scale deletions, and chromosomal translocations [12], accelerating the activation of proto-oncogenes and inactivation of tumor suppressor genes, a process that ultimately drives cancer development [13]. Nevertheless, while the link between folate-mediated one-carbon metabolism and DNA stability is well-established [14], it remains uncertain whether FA deficiency promotes colorectal tumorigenesis specifically through telomere attrition driven by thymidylate synthase-mediated uracil misincorporation.

Current evidence regarding the association between FA supplementation and CRC risk remains inconsistent, and the underlying mechanisms are not fully elucidated. FA appears to influence CRC development through one-carbon metabolism. By supporting the conversion of homocysteine (Hcy) to methionine, FA promotes the synthesis of S-adenosylmethionine (SAM), the major methyl donor for DNA methylation [15]. Dysregulation of this methylation process can contribute to colorectal carcinogenesis by inducing aberrant methylation patterns, such as hypermethylation of tumor suppressor gene promoters (e.g., CDKN2A, MLH1, and APC) and global hypomethylation of proto-oncogenes [16]. Human intervention trials to date suggest that supraphysiological doses of folate can reverse DNA hypomethylation in colorectal mucosa of individuals with colorectal neoplasia [17]. In addition, experimental studies indicate that DNA hypermethylation can suppress MEOX1-mediated GLP2R transcription, activate glycolysis via Hippo pathway inhibition, and thereby promote CRC progression and immune evasion [18]. Although both folate deficiency and excess can disrupt normal DNA methylation [19], the specific roles in modulating metabolic and immune evasion pathways in CRC remain unclear.

Chronic inflammation is a key driver of colorectal carcinogenesis, and elevated levels of inflammatory mediators such as interleukin (IL)-6 and tumor necrosis factor-α (TNF-α) are associated with increased CRC risk and poor prognosis [20]. In addition to its role in one-carbon metabolism, FA may influence CRC development by modulating inflammatory signaling pathways [21]. Experimental evidence further indicates that inflammation-related immune pathways help establish a tumor-promoting microenvironment in the colon [22]. Thus, the effects of FA on CRC risk may extend beyond epigenetic regulation to include inflammation-associated mechanisms, although these interactions remain incompletely understood.

Although folate has been proposed to exert dual effects on colorectal tumorigenesis, it remains unclear whether folate deficiency and excess promote CRC through distinct molecular mechanisms. Previous studies have rarely compared these opposite exposure states within the same experimental model. Using the AOM/DSS mouse model, we therefore investigated the dose-dependent effects of FA and examined whether deficiency- and excess-related CRC risks are associated with uracil misincorporation and aberrant DNA methylation, respectively. This study may help reconcile conflicting evidence on FA and CRC risk and provide insights into more precise prevention strategies.

## 2. Materials and Methods

### 2.1. Animals and Experimental Design

BALB/c male mice (5 weeks old, 22–25 g) were obtained from Jiangsu GemPharmatech Co., Ltd. (Nanjing, China) and housed in a specific pathogen-free environment (a temperature of 23 ± 1 °C, a humidity of 50 ± 5%, 12 h light/dark cycle). All experiments were conducted by following the criteria outlined in the Guide for the Care and Use of Laboratory Animals and were approved by the Tianjin Medical University Animal Ethics Committee (No. TMUaMEC2024031). After a one-week acclimatization, animals showing signs of disease or abnormal behavior, evidence of infection or severe injury, extreme body weight outliers, or deaths unrelated to the experimental protocol were excluded. The remaining mice were randomly divided into five dietary groups (*n* = 15): FA-deficient (FD): <0.1 mg FA/kg diet; FA-normal (FN): 2.0 mg FA/kg diet; low-FA-supplemented (FL): 6.0 mg FA/kg diet; medium-FA-supplemented (FM): 8.0 mg FA/kg diet; and high-FA-supplemented (FH): 20.0 mg FA/kg diet. Randomization was performed using a computer-generated list, stratified by body weight and general health status to ensure balanced baseline characteristics across groups. Diets were obtained from Trophic Animal Feed High-Technology Company (Nantong, China). The FA content in the FN diet (2.0 mg/kg) was formulated according to the AIN-93M standard, which meets the general nutritional requirements for rodents and is considered equivalent to an approximate human intake of 400 μg/d [23]. Based on this physiological baseline, the FA levels in the FL, FM, and FH diets (6.0, 8.0, and 20.0 mg/kg, respectively) were designed to model increasing supplementation states, corresponding to estimated human intakes of 800 μg/d, 1.2 mg/d, and 3.6 mg/d, respectively, as previously described [24,25,26].

**Figure 1:**
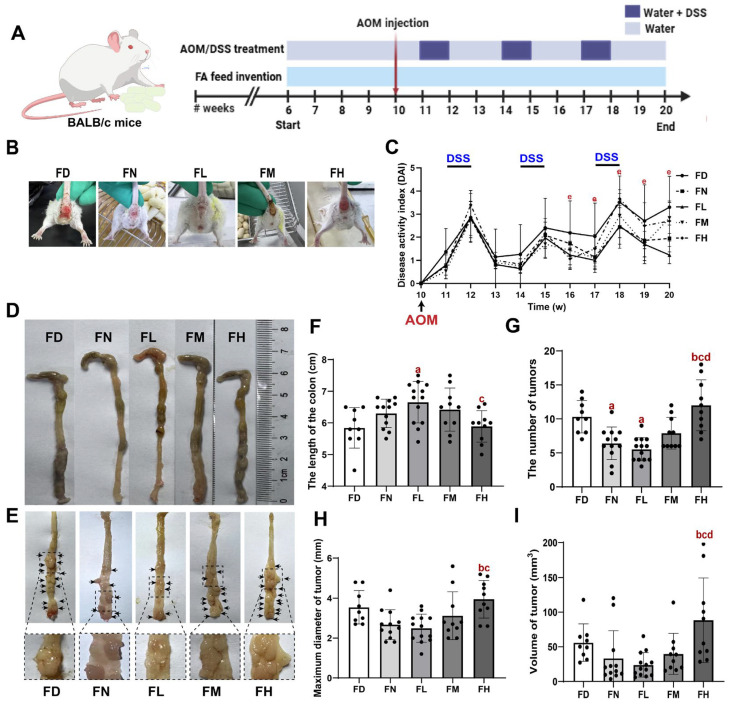
FA deficiency and excess facilitated colorectal carcinogenesis in BALB/c mice. Mice were randomly divided into five groups: folic acid deficiency (FD) group; folic acid normal (FN) group; folic acid low supplementation (FL) group; folic acid medium supplementation (FM) group; folic acid high supplementation (FH) group. The sample sizes for each group were as follows: FD group (n = 9), FN group (n = 12), FL group (n = 13), FM group (n = 10), and FH group (n = 10). (**A**) Schematic diagram showing experimental design and timeline. (**B**) Representative picture of diarrhea. (**C**) Disease activity index. (**D**,**E**) Colonic morphologies. Arrow points at tumor location. (**F**) The length of the colon. (**G**) The number of tumors. (**H**) Maximum diameter of tumor. (**I**) Volume of tumor. Data are presented as mean ±  SD. a: compared to the FD group, *p* < 0.05; b: compared to the FN group, *p* < 0.05; c: compared to the FL group, *p* < 0.05; d: compared to the FM group, *p* < 0.05, e: compared among groups, *p* < 0.05.

As shown in Figure 1A, four weeks after FA intervention during this period, the azomethane/dextran sodium sulfate (AOM/DSS) model was established as previously reported [27]. Briefly, modeling consisted of a single intraperitoneal injection of AOM (10 mg/kg; Sigma-Aldrich, St. Louis, MO, USA), which was freshly dissolved in sterile 0.9% normal saline, filtered through a 0.22 μm filter prior to use, and followed by three cycles of oral administration of 2.5% DSS (MP Biomedicals, Irvine, CA, USA) in drinking water for one week, with each DSS cycle followed by two weeks of normal water. Body weight, diarrhoea, and blood stools were monitored during the modelling process. Disease activity index (DAI) was evaluated during each DSS cycle based on body weight loss, stool consistency, and the presence of fecal occult/gross blood, as previously described [28]. Briefly, scores for body weight loss (0–4), stool consistency (0–4), and bleeding (0–4) were assigned and summed to obtain the DAI for each mouse (Table S1).

At the end of the experiment, mice were euthanized by CO_2_ inhalation in a dedicated chamber at a gradual fill rate of approximately 20–30% of the chamber volume per minute, and death was confirmed by the absence of respiration and reflexes before tissue collection. Blood was then collected by cardiac puncture, processed to obtain serum, aliquoted, and stored at −80 °C until analysis (avoiding repeated freeze–thaw cycles), and colorectal tissues were simultaneously harvested for further examination. The natural length of the colon and rectum was measured, briefly, the entire colon and rectum were carefully dissected from the ileocecal junction to the anus, gently flushed with cold PBS to remove fecal content, and placed flat on a moist, non-stretchable surface without applying tension. The segment was then aligned in a straight line and its length was measured with a calibrated ruler to the nearest millimeter. Subsequently, colorectal tissues were dissected longitudinally, and the number and volume of tumors were recorded. The distal colorectal tissue 1 cm from the anus was dissected and embedded in paraffin, sectioned (3 μm thickness). The remaining portion and the isolated tumor tissue were stored at  −80 °C after liquid nitrogen flash-freezing.

### 2.2. Hematoxylin–Eosin (H&E) Staining

Colorectal tissue sections of each mouse were stained with a HE staining kit (Sparkjade, Jinan, China) according to the manufacturer’s instructions. Briefly, after the processes of dewaxing and dehydration, the sections were stained with hematoxylin for 60 s, followed by exposure to 1% hydrochloric acid ethanol for 3 s, and subsequently stained with eosin for 10 s. After mounting the tissues on slides with coverslips, they were observed using the Olympus IX81 microscope (Olympus, Tokyo, Japan) to evaluate the histological variations.

### 2.3. Immunohistochemistry (IHC)

Paraffin sections of colonic tissue were roasted at 60 °C and then immersed in xylene, gradient ethanol, and ultrapure water. Samples were then incubated overnight at 4 °C with rabbit anti-Ki67 antibody (1:100, Abcam, Cambridge, UK), rabbit anti-proliferating cell nuclear antigen (PCNA) antibody (1:100, Abcam), rabbit anti-p53 antibody (1:100, Abcam) or rabbit anti-thymidine synthase (TS) antibody (1:100, Proteintech, Wuhan, China) after blocked with 10% bovine serum albumin, followed by incubation with secondary antibody for 1 h at room temperature (RT). After staining with DAB working solution, histological changes were observed with an Olympus IX81 microscope and analyzed with ImageJ software (version 1.54, National Institutes of Mental Health, Bethesda, MD, USA).

### 2.4. Quantitative Real-Time Polymerase Chain Reaction (qPCR)

Total RNA was extracted from mouse colorectal tissue using an RNA extraction kit (Sparkjade), purified, quantified with a NanoDrop 2000 (Thermo Fisher Scientific, Waltham, MA, USA), and then reverse transcribed into cDNA. The cDNA was then amplified using gene-specific primers (GenScript, Nanjing, China) and a SYBR Green polymerase reaction kit (Promega Corporation, Madison, WI, USA). The reaction used a 10 μL mixed system: DNA (1 μL, 5 ng/μL), 1 × SYBR Green master mix (Promega) (5 μL), forward primer (0.4 μL, 10 μM), reverse primer (0.4 μL, 10 μM), RNase-free water (3.2 μL). Amplification signals were monitored using a LightCycler 480 II instrument (Roche Applied Science, Basel, Switzerland) under the following conditions: initial denaturation at 95 °C for 5 min, followed by 40 cycles of 95 °C for 30 s and 55 °C for 30 s. The following primers were used: PCNA, forward 5′-GGCTCTCAAAGACCTCATCAA-3′ and reverse 5′-GAGTAAGCTGTACCAAGGAGAC-3′; Ki67, forward 5′-CCTCAAAAGCAGACGAGCAAGA-3′ and reverse 5′-GAGAGTTTGCATGGCCTGTAGT-3′; p53, forward 5′-AATACCAGGGCAACTATGGCTTC-3′ and reverse 5′-AACTGCACAGGGCACGTCTTC-3′; TS, forward 5′-GCTCACAACCAAACGAGTGT-3′ and reverse 5′-TGTCGGGCAGAAAATCCCAA-3′; GAPDH, forward 5′-TGTGTCCGTCGTGGATCTG-3′ and reverse 5′-TTGCTGTTGAAGTCGCAGGAG-3′.

For telomere length analysis, genomic DNA was extracted using a DNA extraction kit (Sparkjade), quantified, and diluted. A standard curve was generated from 12 samples, and the single-copy gene 36B4 was used for normalization. The 10 μL PCR reaction was identical to the above. The cycling conditions were 95 °C for 10 min, followed by 35 cycles of 95 °C for 15 s and 60 °C for 1 min. Telomere PCR was performed using the primers Tel-F 5′-CGGTTTGTTTGGGTTTGGGTTTGGGTTTGGGTTTGGGTT-3′ and Tel-R 5′-GGCTTGCCTTACCCTTACCCTTACCCTTACCCTTACCCT-3′.

For uracil misincorporation analysis in telomeres, genomic DNA was incubated with or without Uracil-DNA Glycosylase (UDG; 1 U/100 ng DNA) at 44 °C for 1 h in buffer (20 mM Tris-HCl, 1 mM EDTA, 1 mM DTT, pH 8.0), followed by enzyme inactivation at 95 °C for 10 min. The PCR reaction and cycling conditions were conducted under identical conditions to the telomere length assay. The difference in Ct values between treated and untreated samples was used to evaluate uracil incorporation levels.

### 2.5. Folate and Hcy Assay

The folate level in serum and colorectal tissue was measured via a competitive protein-binding assay with an automated chemiluminescence system (Siemens immune 2000 xpi, Berlin, Germany) for all types of folates with detection limits of 1–24 ng/mL. The concentration of Hcy in serum and colorectal tissue was assayed using an Auto-Chemistry Analyzer (CS-T300, DIRUI, Changchun, China) with detection limits of 3–50 μmol/L. Protein quantification was assayed using bicinchoninic acid (BCA) protein quantitative Kit (Sparkjade).

### 2.6. Immunofluorescence (IF)

Paraffin sections of mouse colorectal tissue were dewaxed in xylene and dehydrated through a graded alcohol series. Endogenous peroxidase was quenched with 3% H_2_O_2_, followed by permeabilization with 0.5% Triton X-100 and antigen retrieval in citrate buffer at 80 °C. After blocking with 10% goat serum, sections were incubated overnight at 4 °C with the following primary antibodies: rabbit Anti-RAP1 antibody (1:500, Abcam)/Anti-RAP1 GTPase-activating protein (RAP1GAP) antibody (1:50, Proteintech)/Anti-CTLA4 antibody (1:100, CST Danver, Danvers, MA, USA)/Anti-PD-L1 antibody (1:100, CST). A FITC-conjugated goat anti-rabbit secondary antibody (1:100, Sparkjade) was applied, and nuclei were counterstained with 4′, 6-Diamidino-2-Phenylindole (DAPI). Imaging was performed using an Olympus IX81 microscope, and fluorescence signals were quantified with ImageJ software.

### 2.7. Western Blotting (WB)

Tissue lysates were prepared using radioimmunoprecipitation assay (RIPA) buffer (Beijing Beo Tianmei Biotechnology Co., Ltd., Beijing, China), followed by centrifugation at 14,000 *g* for 10 min at 4 °C to collect proteins. Protein concentrations were measured using the BCA Protein Assay Kit. A total of 20 μg of protein per sample was separated by 10% dodecyl sulfate, sodium salt (SDS), and polyacrylamide gel electrophoresis and transferred onto a PVDF membrane (Millipore, Schwalbach, Germany) by wet electrotransfer. The membrane was then blocked with 5% skimmed milk (Sigma-Aldrich, St. Louis, MO, USA) and 1× Tris buffered saline Tween for 1 h at RT, followed by primary rabbit monoclonal antibody (anti-TS (1:1000, CST), anti-cytotoxic T-Lymphocyte-Associated protein 4 (CTLA4) (1:1000, CST), anti-programmed death-ligand 1 (PD-L1) (1:1000, CST), anti-RAP1 (1:5000, Abcam), anti-RAP1 GTPase-activating protein (RAP1GAP) (1:1000, Abcam) at 4 °C overnight. The membranes were subsequently incubated with secondary antibodies (horseradish peroxidase-conjugated anti-rabbit IgG, 1:1000, CST) for 1 h at 25 °C. The blots were detected by enhanced chemiluminescent substrates (SparkJade) and observed with the ChemiDocTM XRS+ Imaging System (Bio-Rad, Hercules, CA, USA). Densitometric analysis was used to quantify the protein levels with ImageJ Software.

### 2.8. Telomere Attrition Assay

Telomere length and telomeric DNA damage were detected via Immunofluorescence-Fluorescent In Situ Hybridization (IF-FISH) in colorectal tissues using a fluorescent peptide nucleic acid (PNA) (CCCTAACCCTAACCCTAA; F1002; PNA Bio, Thousand Oaks, CA, USA). Following deparaffinization, rehydration, and antigen retrieval in citrate buffer, sections were permeabilized with 0.5% Triton X-100 and dehydrated through an ethanol series. Hybridization was performed with the telomere PNA probe under denaturing conditions (85 °C, 5 min), followed by overnight incubation at 4 °C. After stringency washes, samples were blocked with 10% goat serum and stained with rabbit anti-p53-Binding Protein 1 (53BP1) antibody (1:300, Abcam, 4 °C, overnight) and FITC-conjugated goat anti-rabbit secondary antibody (1:100, SparkJade). Nuclei were counterstained with DAPI. Images were acquired using an Olympus IX81 microscope (100× objective). Telomere signal intensity was quantified as PNA signal normalized to DAPI fluorescence using the ImageJ plugin Telometer. Co-localization of telomeric signal (TelC-FITC) with 53BP1 foci was defined as telomere dysfunction-induced foci (TIF)-positive, with ≥2 foci per nucleus considered positive [29].

### 2.9. High-Performance Liquid Chromatography (HPLC)

The concentrations of deoxyuridine monophosphate (dUMP) and deoxythymidine monophosphate (dTMP) in colorectal tissue were quantified using HPLC. Tissue samples (15 mg) were homogenized in 300 μL of 0.1 M ice-cold perchloric acid and centrifuged at 20,000× *g* for 20 min. Subsequently, 250 μL of the supernatant was alkalized with 62.5 μL of 0.5 mol/L sodium carbonate, and analyzed on a Waters HPLC system (Milford, MA, USA) equipped with a Hypersil GOLDTM aq column (Thermo Fisher Scientific). Isocratic elution was performed at 0.26 mL/min and 24 °C using a mobile phase of 0.005 mol/L KH_2_PO_4_ (pH 2.5) containing 0.5% methanol. Analytes were detected at 260 nm, and dUMP/dTMP were quantified by comparison with authentic standards (Sigma-Aldrich, St. Louis, MO, USA).

### 2.10. Whole-Genome Bisulfite Sequencing (WGBS) and Bioinformatics Analysis

WGBS was performed on three colorectal tissue samples from each of the FN and FH groups. Genomic DNA underwent quality control and library preparation as follows: DNA was fragmented into 100–600 bp using a Covaris ME220 sonicator (Covaris, Woburn, MA, USA), followed by blunt-ending, 3′-dA tailing, and adapter ligation with methylated adapters to prevent bisulfite-induced degradation. The adapter-ligated DNA was purified using MagicPure Size Selection DNA Beads (TransGen Biotech, Beijing, China) and bisulfite-converted with the EZ DNA Methylation-Gold Kit (Zymo Research, Irvine, CA, USA). The converted libraries were amplified for 8–10 cycles using NEBNext^®^ Q5U^®^ Master Mix (New England Biolabs, Ipswich, MA, USA), quantified with a Qubit fluorometer (Thermo Fisher Scientific), and size-verified. Equimolar pools were sequenced on an Illumina Nova platform (Illumina, San Diego, CA, USA) in 2 × 150 bp paired-end mode.

Raw FastQ files were processed for quality control using FastQC v0.11.8 (Babraham Bioinformatics, Cambridge, UK) and custom R scripts to remove adapters, trim low-quality bases, and exclude duplicates. Clean reads were aligned to the reference genome, and uniquely mapped high-quality reads were used for downstream analysis. Differential methylation analysis was conducted with methylKit v1.24.0 (https://bioconductor.org/packages/methylKit/, accessed on 4 January 2026); resulting DMRs/DMCs were annotated using ANNOVAR (version 2018Apr16; https://annovar.openbioinformatics.org/, accessed on 4 January 2026). Functional enrichment and pathway analyses were performed with clusterProfiler v4.6.2 (https://bioconductor.org/packages/clusterProfiler/, accessed on 4 January 2026).

### 2.11. Enzyme-Linked Immunosorbent Assay (ELISA)

The levels of inflammatory cytokines such as TNF-α, IL-6, IL-1β, IL-10, transforming growth factor-β (TGF-β) in the colorectal tissue were measured using standard ELISA kits (MEIMIAN, Jiangsu, China) following the manufacturer’s instructions. Protein quantification was assayed using a BCA protein quantitative kit.

### 2.12. Statistical Analysis

Data are presented as mean ± standard deviation (SD). Differences among multiple groups were analyzed by one-way analysis of variance (ANOVA), and repeated measures ANOVA was used for multi-time-point comparisons. Post hoc multiple comparisons were conducted using the Student–Newman–Keuls (SNK) method when significant differences were detected. Comparisons between two groups were performed using an unpaired two-tailed Student’s *t*-test. Survival analysis was performed with the Log-rank test. Statistical analyses were carried out using SPSS 24.0 (IBM Corp, Armonk, NY, USA), with *p* < 0.05 considered statistically significant. GraphPad Prism 8.0.1 software (GraphPad Software, La Jolla, CA, USA) was used to make charts.

## 3. Results

### 3.1. FA Deficiency and Excess Facilitated Colorectal Carcinogenesis in BALB/c Mice

To evaluate the impact of FA levels on CRC development, we employed the AOM/DSS-induced CRC model in mice. As shown in Figure 1, mice in the FD and FH groups exhibited prolapse accompanied by severe diarrhea and hematochezia, while symptoms were reduced in the FN and FM groups, with the FL group showing the mildest symptoms. No statistically significant differences in survival probability were observed among the groups (*p* > 0.05, Figure S1).

Temporal changes in DAI scores were presented in Figure 1C. Initially, the DAI score was 0. Following intraperitoneal injection of AOM, the DAI score increased. Subsequently, each round of DSS drinking water administration further elevated the mice’s scores, which then decreased after switching to normal drinking water. A significant divergence was observed between 19 and 20 weeks of age, during which the FL group’s DAI score decreased while scores in the other four groups increased (*p* < 0.05).

Compared to the other four groups, the FL group exhibited the longest colorectal length, the fewest tumors, the smallest maximum tumor diameter, and the lowest tumor volume, followed by the FN and FM groups. In contrast, the FD and FH groups displayed the most severe outcomes (*p* < 0.05, Figure 1D–I). Therefore, these findings suggested that both insufficient and excessive FA intake promoted the development of AOM/DSS-induced CRC in mice, whereas moderate FA supplementation exerted a protective effect.

### 3.2. FA Deficiency and Excess Induced Cell Proliferation in Colorectal Tissues of BALB/c Mice

**Figure 2:**
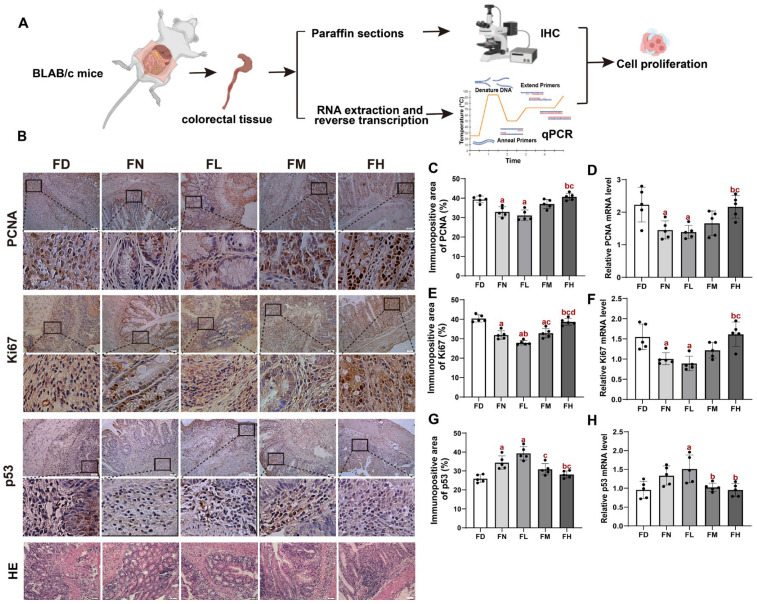
FA deficiency and excess induced cell proliferation in colorectal tissues of BALB/c mice. Mice were assigned to intervention groups as described in Figure 1. (**A**) Schematic diagram of H&E and IHC staining. (**B**) Representative images of IHC and H&E staining in colorectal tissues. Scale bar = 50 μm. Each column below the merged image shows an enlarged image of the corresponding image rectangle on the merged image. (**C**) Immunopositive area of PCNA. (**D**) Relative PCNA mRNA level. (**E**) Immunopositive area of Ki67. (**F**) Relative Ki67 mRNA level. (**G**) Immunopositive area of p53. (**H**) Relative p53 mRNA level. Data are presented as mean ±  SD (n = 5). a: compared to the FD group, *p* < 0.05; b: compared to the FN group, *p* < 0.05; c: compared to the FL group, *p* < 0.05; d: compared to the FM group, *p* < 0.05.

To further evaluate the impact of FA levels on colorectal carcinogenesis, we evaluated cell proliferation in colorectal tissues from BALB/c mice using IHC and qPCR (Figure 2A). Both analyses revealed that the proliferation markers PCNA and Ki67 exhibited the lowest protein and mRNA expression levels in the FL group, followed by the FN and FM groups. In contrast, higher expression levels were observed in the FH and FD groups (*p* < 0.05, Figure 2B–F). Consistently, tumor suppressor gene p53 levels exhibited an opposite trend, with the highest levels in the FD and FH groups and the lowest in the FL group (*p* < 0.05, Figure 2B,G,H). H&E staining and histological examination confirmed that AOM/DSS induction resulted in typical CRC features, including distorted crypt architecture and severe mucosal damage. These pathological alterations were markedly attenuated by adequate FA intake (FL group) but exacerbated under both FA-deficient and FA-excess conditions (Figure 2G). Together, these results further support the conclusion that both insufficient and excessive FA intake promote the development of AOM/DSS-induced colorectal carcinogenesis in mice.

### 3.3. Serum and Colorectal Tissue Folate and Hcy Levels in BALB/c Mice

**Figure 3:**
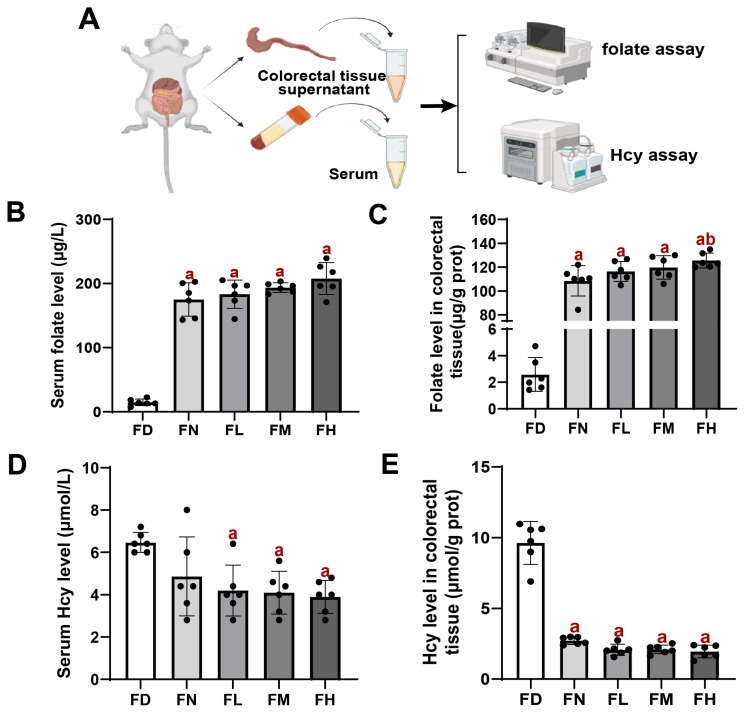
FA deficiency reduced serum and colorectal tissue folate levels and increased Hcy levels in BALB/c mice. Mice were assigned to intervention groups as described in Figure 1. (**A**) Schematic diagram of folate and Hcy assay. (**B**) Serum folate level. (**C**) Folate level in colorectal tissue. (**D**) Serum Hcy level. (**E**) Hcy level in colorectal tissue. Data are presented as mean ±  SD (n = 6). a: compared to the FD group, *p* < 0.05. b: compared to the FN group, *p* < 0.05.

Folate and Hcy levels were measured in both serum and colorectal tissues of mice. As expected, the FD group showed the lowest folate concentrations in both serum and colorectal tissue compared to the other four groups (*p* < 0.05, Figure 3B, C). Conversely, Hcy levels were highest in the FD group and decreased progressively with increasing FA intake (*p* < 0.05, Figure 3E, F). These findings suggest that FA deficiency leads to a reduction in folate concentrations in both mouse colorectal tissue and serum, while simultaneously elevating Hcy levels. Increasing FA intake resulted in an upward trend in both serum and colorectal tissue folate levels, accompanied by a corresponding decrease in Hcy concentrations.

### 3.4. FA Deficiency May Facilitate Colorectal Carcinogenesis by Uracil Misincorporation-Mediated Telomere Attrition in BALB/c Mice

**Figure 4:**
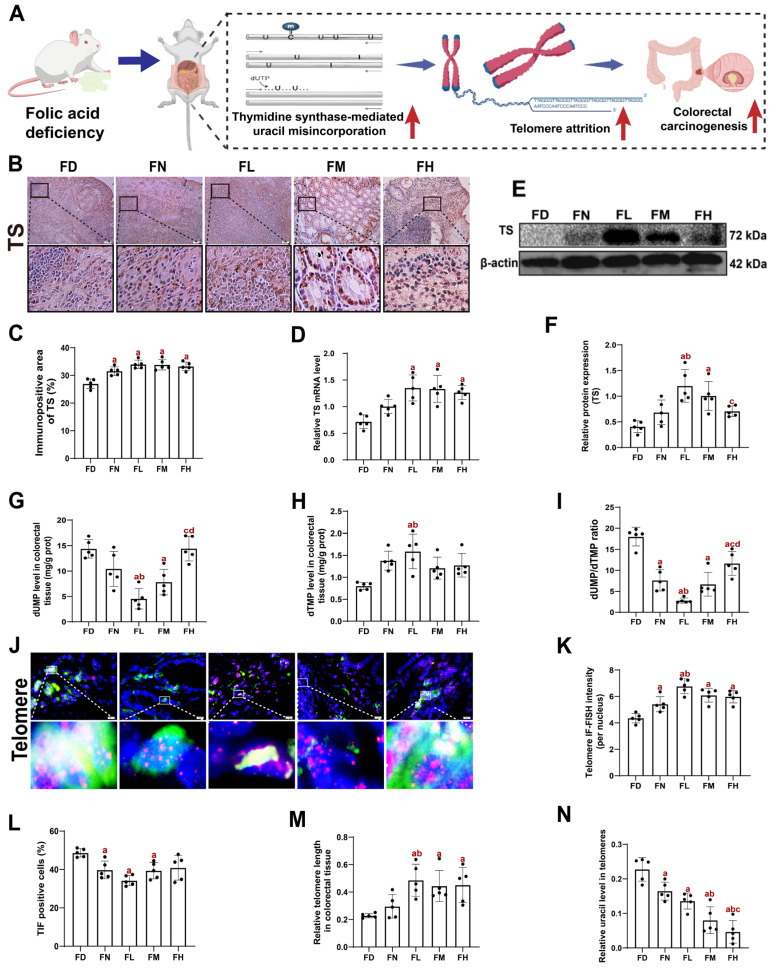
FA deficiency may facilitate colorectal carcinogenesis by uracil misincorporation-mediated telomere attrition in BALB/c mice. Mice were assigned to intervention groups as described in Figure 1. (**A**) Schematic diagram of the mechanism of FA deficiency affecting colorectal carcinogenesis in BALB/c mice. The red arrow indicates an increase in the indicator by FA deficiency (**B**) Representative images of IHC staining of TS in colorectal tissue. Scale bar = 50 μm. Each column below the merged image shows an enlarged image of the corresponding image rectangle on the merged image. (**C**) Immunopositive area of TS. (**D**) Relative TS mRNA level. (**E**) Representative Western blots of TS. (**F**) Densitometric analysis of TS. (**G**) dUMP levels in the colorectal tissue. (**H**) dTMP levels in the colorectal tissue. (**I**) dUMP/dTMP ratio in the colorectal tissue. (**J**) Representative images of IF-FISH of colorectal tissue, in which cells are stained with 53BP1 (green), telomere (red), and DAPI (blue). Scale bar = 10  μm. Each column below the merged image shows an enlarged image of the corresponding image rectangle on the merged image. (**K**) Telomere length quantified by IF-FISH in colorectal tissue. (**L**) Percentages of TIF cells. (**M**) Relative telomere length in colorectal tissue. (**N**) Relative uracil levels in telomeres. Data were presented as mean ± SD (n = 5). a: compared to the FD group, *p* < 0.05; b: compared to the FN group, *p* < 0.05; c: compared to the FL group, *p* < 0.05; d: compared to the FM group, *p* < 0.05.

To investigate the potential mechanisms by which FA deficiency affects the development of CRC, we first employed IHC, qPCR, and WB analysis to detect TS protein and mRNA levels in mouse colorectal tissues. Our results revealed that TS protein and mRNA levels were higher in the FL group and lower in the FD group (*p* < 0.05, Figure 4B–F). However, no consistent increase in TS levels was observed in the FM and FH groups. These findings indicated that FA deficiency reduced TS expression in mouse colorectal tissues, pointing to its involvement in FA-related tumorigenesis.

TS expression is closely linked to DNA synthesis, and reduced TS activity results in the erroneous incorporation of uracil into DNA [30]. In the present study, dUMP levels in colorectal tissue were lower in the FL group compared to the FD and FN groups, while dTMP levels exhibited the opposite trend *(p* < 0.05, Figure 4K–M). Notably, with increasing FA supplementation, dUMP levels showed a gradual increase, whereas dTMP levels steadily decreased. Correspondingly, the dUMP/dTMP ratio in the five groups followed a similar trend to that observed for dUMP levels. HPLC analysis indicated that FA deficiency increased uracil misincorporation in mouse colorectal tissues, likely driven by reduced TS activity.

Increased uracil incorporation leads to heightened DNA damage, which disrupts the “cap-like” protective structure of telomeres, resulting in telomere dysfunction [31]. IF-FISH and qPCR analyses revealed that telomere length in colorectal tissue was shortest in the FD group, followed by the FN group, with the FL group displaying the longest telomeres (*p* < 0.05, Figure 4H,J). However, telomere length did not increase consistently with higher FA supplementation beyond the FL group. Correspondingly, the number of TIF-positive cells was highest in the FD group, decreased in the FN group, and was lowest in the FL group, but rose again in the FM and FH groups (*p* < 0.05, Figure 4I). The relative level of uracil in telomeres of the FD group was the highest among all groups and declined progressively with increasing FA intake (*p* < 0.05, Figure 4N). These data further support the role of FA deficiency in promoting uracil misincorporation and telomere attrition, contributing to colorectal carcinogenesis.

In summary, these findings suggest that FA deficiency promotes CRC development in mice by inducing telomere attrition through TS-mediated uracil misincorporation. The non-corresponding responses in the FM and FH groups suggest that alternative mechanisms may operate under conditions of FA excess.

### 3.5. FA Excess May Facilitate Colorectal Carcinogenesis by Tumor Immunosuppression Mediated Through RAP1GAP Hypermethylation in BALB/c Mice

**Figure 5:**
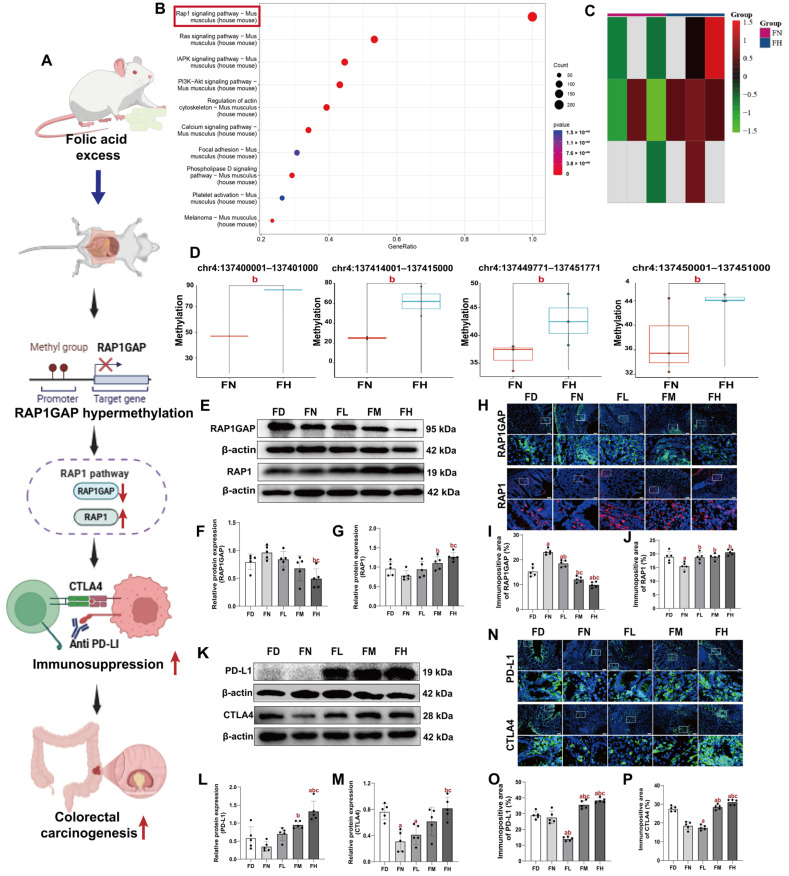
FA excess may facilitate colorectal carcinogenesis by tumor immunosuppression mediated through *RAP1GAP* hypermethylation in BALB/c mice. Mice were assigned to intervention groups as described in Figure 1. (**A**) Schematic diagram of the mechanism of FA excess affecting colorectal carcinogenesis in BALB/c mice. An upward red arrow indicates an increase in the parameter, whereas a downward red arrow indicates a decrease. (**B**) KEGG pathway analysis of DMGs. The ordinate represents the enriched pathways, and the abscissa represents the rich factor of the corresponding pathways; the size of the spots represents the number of genes related to DMR enriched in each pathway. (**C**) Heatmap analysis of methylation levels of DMGs in the RAP1 signaling pathway. (**D**) RAP1 signaling pathway-related methylation levels of DMGs. (**E**,**K**) Representative Western blots of RAP1GAP, RAP1, CTLA-4 and PD-L1. (**F**,**G**,**L**,**M**) Densitometric analysis of RAP1GAP, RAP1, CTLA-4, and PD-L1. (**H**,**N**) Representative images of IF, in which cells are stained with RAP1GAP/CTLA-4/PD-L1 (green), RAP1 (red) and DAPI (blue). Scale bar = 50  μm. Each column below the merged image shows an enlarged image of the corresponding image rectangle on the merged image. (**I**,**J**,**O**,**P**) Immunopositive area of RAP1GAP, RAP1, CTLA-4 and PD-L1. Data were presented as mean ±  SD (n = 3/5). a: compared to the FD group, *p* < 0.05. b: compared to the FN group, *p* < 0.05; c: compared to the FL group, *p* < 0.05.

To elucidate the potential molecular mechanisms through which excessive FA promotes colitis-associated CRC, we performed WGBS to assess genome-wide DNA methylation patterns in colorectal tissues from mice in the FN and FH groups. To explore the biological functions of differentially methylated genes (DMGs) within the upstream 2 kb region, we conducted KEGG pathway enrichment analysis comparing DMGs between the two groups. The analysis revealed that these DMGs are predominantly enriched in key signaling pathways, including RAP1, Ras, MAPK, and PI3K-Akt signaling, all of which play crucial roles in the initiation and progression of CRC (Figure 5B). Among these pathways, the RAP1 signaling pathway emerged as the most significantly enriched. As it remains unclear whether DNA methylation directly regulates this pathway, we focused our subsequent analysis on its epigenetic regulation.

Cluster heatmap analysis of methylation levels of DMGs in the Rap1 GTPase-activating protein (RAP1GAP) signaling pathway showed that most of these loci were hypermethylated in the FH group, whereas hypomethylated DMGs constitute a smaller fraction (Figure 5C). Methylation levels at specific differentially methylated regions (DMRs) within the RAP1 gene are displayed in Figure 5D. Significantly higher methylation was observed in the FH group compared to the FN group at the following genomic regions: chr4:137400001–137401000, 137449771–137451771, 137414001–137415000, and 137450001–137451000 (*p* < 0.05, Figure 5D).

To validate the results, we employed WB and IHC to assess RAP1GAP protein expression levels. The findings revealed that the FN group exhibited the highest RAP1GAP protein expression, which progressively decreased with FA supplementation. The FD group showed slightly lower expression levels compared to the FN group (*p* < 0.05, Figure 5F,L). Consistent with the established role of RAP1GAP in suppressing RAP1 activity [32], reduced RAP1GAP expression in our models correlated with upregulated RAP1 activity across the experimental groups (*p* < 0.05, Figure 5G,M).

Existing evidence suggests that the RAP1 pathway is closely linked to immune function in tumor cells [33]. We separately employed WB analysis to detect the expression levels of immune checkpoint proteins PD-L1 and CTLA-4 in mouse colorectal tumor tissues. IF was used to examine the protein expression of PD-L1 and CTLA-4 within the tumor microenvironment of mouse colorectal tissues. Consistently, the results demonstrated that expression of PD-L1 and CTLA-4 was lower in the FN group than in the FM and FH groups (*p* < 0.05, Figure 5I, J, O, P). However, expression patterns of these proteins were inconsistent between the FD and FL groups.

These findings collectively indicated that excessive FA intake promoted colorectal tumorigenesis by activating RAP1 through RAP1GAP hypermethylation and thereby facilitating an immunosuppressive microenvironment. However, FA deficiency appeared to drive CRC progression through mechanisms independent of this pathway.

### 3.6. Effects of FA Deficiency and Excess on Inflammatory Factors in the Colorectal Tissue of BALB/c Mice

**Figure 6:**
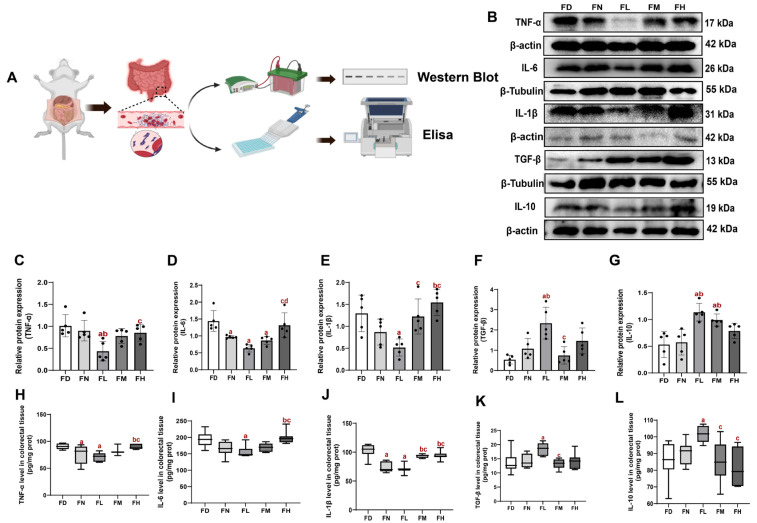
Effects of FA status on inflammatory factors in colorectal tissue of BALB/c mice. Mice were assigned to intervention groups as described in Figure 1. (**A**) Schematic diagram of Inflammatory factors assay. (**B**) Representative Western blots of TNF-α, IL-6, IL-1β, TGF-β, and IL-10. (**C**–**G**) Densitometric analysis of TNF-α, IL-6, IL-1β, TGF-β, and IL-10 (n = 5). (**H**–**L**) TNF-α, IL-6, IL-1β, TGF-β and IL-10 levels in colorectal tissue by ELISA (n = 7). Data are presented as mean ± SD. a: compared to the FD group, *p* < 0.05. b: compared to the FN group, *p* < 0.05; c: compared to the FL group, *p* < 0.05; d: compared to the FM group, *p* < 0.05.

To further validate these findings, we quantified the levels of key inflammatory factors (TNF-α, IL-1β, IL-6, IL-10, and TGF-β) in mouse colorectal tissues using WB and ELISA. The levels of proinflammatory cytokines (TNF-α, IL-1β, and IL-6) were lowest in the FL group, followed by the FN and FM groups, and highest in the FD and FH groups (*p* < 0.05, Figure 6 C–E, H–J). Collectively, these data reveal a U-shaped relationship between FA intake and colorectal pro-inflammatory responses, with both deficiency and excess promoting inflammation.

IL-10 and TGF-β are known to cooperatively establish an immunosuppressive microenvironment that facilitates tumor immune evasion by suppressing effector immune cell function [34]. In our study, WB and ELISA showed that TGF-β levels were highest in the FL group, while the FD, FM and FH groups had lower levels (*p* < 0.05, Figure 6F, K), indicating a non-linear response of TGF-β to FA status. Although TGF-β in FH was slightly higher than in FM, this did not parallel tumor burden, suggesting that TGF-β alone cannot explain FA-dependent differences in tumor growth. IL-10 levels were similarly higher in FL and were reduced in FD, FM and FH (*p* < 0.05, Figure 6G, L), indicating that both FA deficiency and excess disrupt the anti-inflammatory cytokine milieu.

## 4. Discussion

CRC originates from the epithelial lining of the colon or rectum, and its pathogenesis involves a complex, multistage process driven by the accumulation of genetic and epigenetic alterations that promote tumor initiation and progression [35]. Although epidemiological and animal studies have long suggested a link between FA intake and CRC risk, the nature of this association remains incompletely defined. In this study, we used an AOM/DSS-induced CRC model in BALB/c mice fed graded levels of FA to clarify its dose-dependent effects. Our findings indicated that dietary FA might exhibit a dual, non-linear (U-shaped) relationship with colorectal carcinogenesis, whereby both deficiency (<0.1 mg/kg) and excess (8.0/20.0 mg/kg) increased tumor burden—characterized by higher tumor number, larger size, greater volume, shortened colorectal length, and enhanced cell proliferation—through distinct mechanisms, with FA deficiency associated with TS downregulation, dUMP accumulation, dTMP depletion, uracil misincorporation, and telomere attrition, and excessive FA intake associated with RAP1GAP hypermethylation, reduced Rap1GAP expression, enhanced RAP1 activity, and PD-L1/CTLA4 upregulation-mediated immunosuppression, whereas an intermediate intake level (6.0 mg/kg) conferred optimal protection.

Inverse associations between total folate intake and CRC risk have been consistently reported in epidemiological studies and summarized in meta-analyses [4,7]; however, the specific protective dosage of FA remains to be elucidated. A recent analysis of the NHANES dataset by Aglago EK et al. suggested that a total daily folate intake of ≥500 µg dietary folate equivalents (DFE), from both food and supplements, was associated with an 88% reduction in CRC mortality risk [36]. Similarly, in this large prospective cohort with 8.5 years of post-fortification follow-up, high total folate intake (≥900 μg DFE/d) was associated with a 30% lower CRC risk, regardless of fortification period [37]. These observations are corroborated by experimental studies; for example, high-dose FA supplementation was shown to suppress 1,2-dimethylhydrazine (DMH)-induced CRC in ICR mice [38]. Consistently, our study demonstrated that adequate FA intake—corresponding to approximately 800 µg/d FA intake in humans—conferred the most pronounced protective effect against AOM/DSS-induced colorectal tumorigenesis in mice.

A key mechanism by which folate influences carcinogenesis is through the maintenance of genomic stability. It plays a fundamental role in maintaining genomic stability by supporting key processes, including gene expression, nucleotide biosynthesis, DNA repair, and epigenetic regulation, serving as an essential cofactor in purine and thymidylate synthesis [39,40]. In the present study, FA deficiency in mice reduced TS activity, elevated uracil misincorporation in colorectal tissues, and enhanced carcinogenesis, effects which were suppressed by adequate FA intake. FA deficiency has also been demonstrated to disrupt telomere maintenance and integrity [31]. Based on our prior evidence linking uracil misincorporation to telomere attrition [12], we assessed telomere function in the current model. FA deficiency induced telomere attrition, manifested as shortened telomere length and increased telomere damage. This adverse phenotype was reversed upon adequate FA intake (FL group), whereas higher FA doses conferred no additional protective benefit. Taken together, these findings support the conclusion that FA deficiency may exert malignant effects on mouse colorectal carcinogenesis through TS-mediated increased uracil incorporation, leading to telomere attrition.

Beyond the well-established risks of FA deficiency, the potential impact of excessive FA intake on CRC has attracted increasing scientific interest. This concern is particularly relevant in countries like the United States and Canada, where mandatory FA fortification of grain products since 1998 has successfully elevated population folate levels to prevent neural tube defects [41]. Although epidemiological studies reported no increased CRC risk associated with high FA intake during the post-fortification era [42], the public health implications remain a subject of ongoing investigation. Our recent global analysis of FA fortification policies in 193 WHO member states identified an association between mandatory fortification and a lower incidence of cancers of the colon and rectum [43]. Nevertheless, the current experimental findings demonstrated that supra-nutritional doses of FA can promote colorectal carcinogenesis in mice, a result that aligns with the earlier conclusions of Kim YI et al. [44].

Based on this, we further investigated the underlying mechanisms by which supraphysiological FA intake influences colorectal carcinogenesis. As a central methyl donor in one-carbon metabolism, folate influences both global and gene-specific methylation patterns [45]. Excessive FA supplementation has been linked to aberrant methylation patterns, which may adversely affect CRC progression [46]. In the present study, WGBS analysis revealed that excessive FA intake resulted in hypermethylation of the RAP1GAP gene in colorectal tissues of mice. Rap1GAP has been demonstrated to play a crucial role by specifically activating Rap1, functioning as a key negative regulator of RAP1 activity in CRC [47]. Consistent with the methylation data, WB and IF analyses showed reduced RAP1GAP expression and concomitant elevation of RAP1 protein levels in both medium- and high-dose FA groups (FM and FH groups) compared with the normal-intake group. Given reported connections between RAP1GAP downregulation, RAP1 activation, and altered immune signaling in the tumor microenvironment [48], we evaluated the expression of immune checkpoint molecules PD-L1 and CTLA-4. Our results indicated that FA excess enhanced PD-L1 and CTLA-4 levels, suggesting a potential mechanism of immunosuppression facilitated by RAP1 pathway activation. In summary, these results suggested that excessive FA may promote AOM/DSS-induced CRC in mice through RAP1GAP promoter hypermethylation, subsequent Rap1 dysregulation, and ultimately, tumor-induced immunosuppression.

Substantial epidemiological and experimental evidence has established chronic inflammation as a major risk factor for CRC development [49]. The AOM/DSS model employed in our study, as a well-established and widely used experimental system, effectively replicates the pathogenesis of human inflammation-associated colorectal cancer by combining mutagenesis induction with periodic inflammatory stimulation [50]. Our study demonstrates that both deficient and excessive FA intake elevated pro-inflammatory cytokine levels and suppressed anti-inflammatory mediators in mouse colorectal tissue, whereas adequate FA intake mitigated these inflammatory responses, consistent with prior evidence linking folate status to inflammatory regulation [51,52]. These findings suggested that FA imbalance promoted colorectal carcinogenesis, at least in part, through dysregulation of the local inflammatory microenvironment [53]. Especially, TGF-β expression was slightly elevated in the FH group compared to the FM group. Given TGF-β’s established role in immunosuppression [54], this points to a potential association between high FA exposure and enhanced immune suppression in colorectal tissue. This immune-editing mechanism may represent an alternative pathway through which excessive FA facilitates tumor progression under inflammatory conditions.

This study revealed a U-shaped relationship between FA intake and colorectal tumorigenesis, with both deficient and excessive FA intake exerting detrimental effects, in line with epidemiological and experimental data suggesting that folate may have dose-dependent and context-dependent effects on colorectal cancer risk [6,7,8]. Importantly, an adequate FA intake level corresponding to approximately 800 μg/d in humans provided maximal protection [26], underscoring the importance of maintaining FA levels within a strictly defined physiological range. Mechanistically, FA deficiency was found to promote genomic instability through uracil misincorporation and telomere attrition, whereas supra-nutritional FA levels potentially accelerated the progression of abnormal gene methylation-mediated immunosuppression. These findings provide novel insights into the dose-dependent dual role of FA in CRC, offering crucial evidence for refining nutritional strategies for cancer prevention and treatment.

Collectively, our data support a non-linear role of folate in colorectal carcinogenesis, with both deficiency and excess exerting potentially deleterious effects through distinct molecular routes. Folate deficiency is associated with impaired dTMP synthesis, uracil misincorporation, and genomic instability [55], whereas folate excess may induce epigenetic alterations, including site-specific DNA hypermethylation, and simultaneously provide substrates for accelerated tumor cell proliferation [56,57]. This dual role highlights the complexity of folate in the biology of CRC, and future research should focus on identifying dose thresholds and the mechanisms by which folate shifts from maintaining genomic stability to promoting tumor progression, particularly in the context of one-carbon metabolism and epigenetic regulation.

Finally, several limitations of this study should be acknowledged. Although chemically induced rodent models recapitulate key features of human CRC, important interspecies differences remain, and the FA dosages and intervention duration used in this study may not fully reflect typical human exposure patterns. In addition, the molecular mechanisms underlying the effects of FA imbalance were only partially elucidated and require further in-depth investigation. Future studies should therefore focus on developing optimized human research protocols to define the appropriate dosage and timing of folate intake for CRC prevention, thereby improving the safety and translational relevance of folate-based interventions. Furthermore, research leveraging gene-editing technologies is warranted to decipher how FA deficiency promotes uracil misincorporation and how FA excess orchestrates hypermethylation-driven carcinogenesis.

## 5. Conclusions

Our findings demonstrate that both a deficiency in and an excess of FA promote CRC development in AOM/DSS-induced BALB/c mice, whereas adequate FA intake exerts a protective effect. The underlying mechanisms appear to differ: FA deficiency may facilitate colorectal carcinogenesis through uracil misincorporation-mediated telomere attrition, while FA excess can promote carcinogenesis via Immunosuppression mediated by RAP1GAP hypermethylation. This study not only delineates the contrasting mechanisms of folate-driven carcinogenesis but also underscores the pivotal role of optimal FA intake in CRC prevention.

## Data Availability

The original contributions presented in this study are included in the article/Supplementary Material. Further inquiries can be directed to the corresponding authors.

## Supplementary Materials

The following supporting information can be downloaded at https://www.mdpi.com/article/10.3390/nu18081187/s1: Table S1. Disease activity index (DAl). Figure S1. Survival probability for each group.

## Abbreviations

The following abbreviations are used in this manuscript:

FA	folic acid
CRC	colorectal cancer
Hcy	homocysteine
AOM	azoxymethane
DSS	dextran sulfate sodium
DAI	disease activity index
TS	thymidylate synthase
RAP1	Ras-associated protein 1
RAP1GAP	RAP1 GTPase-activating protein
PD-L1	programmed death-ligand 1
CTLA-4	cytotoxic T-lymphocyte-associated protein 4
dUMP	deoxyuridine monophosphate
dTMP	deoxythymidine monophosphate
dUTP	deoxyuridine triphosphate
TIF	telomere dysfunction-induced foci
53BP1	p53-binding protein 1
HPLC	high-performance liquid chromatography
IHC	immunohistochemistry
WB	Western blot
qPCR	quantitative polymerase chain reaction
RT-qPCR	reverse transcription quantitative polymerase chain reaction
IF	immunofluorescence
IF-FISH	immunofluorescence-fluorescence in situ hybridization
WGBS	whole-genome bisulfite sequencing
TNF-α	tumor necrosis factor-α
IL-1β	interleukin-1β
IL-6	interleukin-6
IL-10	interleukin-10
TGF-β	transforming growth factor-β
DAPI	4′,6-diamidino-2-phenylindole
RIPA	radioimmunoprecipitation assay
ANOVA	analysis of variance
SNK	Student–Newman–Keuls
SD	standard deviation
