# Supplementary material for: Deficiency and Excess of Folic Acid Intake Promote Colorectal Carcinogenesis in AOM/DSS-Treated Mice: Roles in Uracil Misincorporation and DNA Methylation
Source: Nutrients. 2026 Apr 9;18(8):1187. doi: 10.3390/nu18081187 (PMC13118905; doi:10.3390/nu18081187)
Supplement: Supplementary file 1 [file nutrients-18-01187-s001.zip › nutrients-4217931-supplementary.pdf]

Supplementary Materials

Table S1. Disease activity index (DAI).

Index	Weight loss (%)	Stool	Crypt damage
0	None	Well-formed pellets	None
1	1-5	-	-
2	6-10	Pasty and semiformed	Positive bleeding
3	11-20	-	-
4	>20	Liquid	Gross bleeding

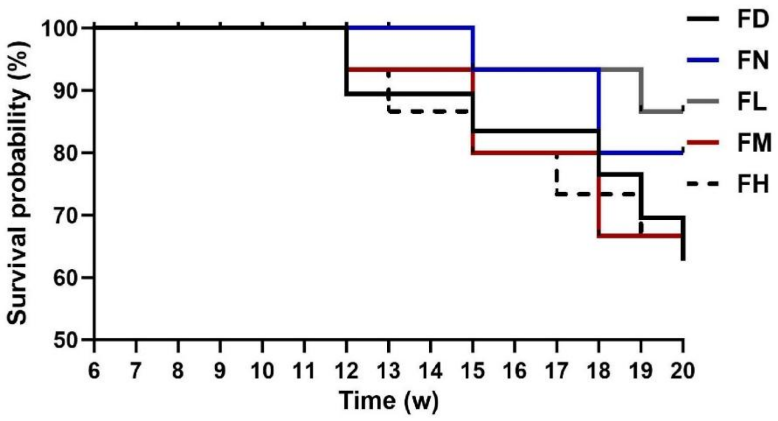

Figure S1. Survival probability for each group (n = 15).

Table S2. RT-qPCR primer sequences.

Gene	Forward primer	Reverse primer
PCNA	GGCTCTCAAAGACCTCATCAA	GAGTAAGCTGTACCAAGGAGAC
Ki67	CCTCAAAAGCAGACGAGCAAGA	GAGAGTTTGCATGGCCTGTAGT
p53	AATACCAGGGCAACTATGGCTTC	AACTGCACAGGGCACGTCTTC
TS	GCTCACAACCAAACGAGTGT	TGTCGGGCAGAAAATCCCAA
GAPHD	TGTGTCCGTCGTGGATCTG	TTGCTGTTGAAGTCGCAGGAG
Tel	CGGTTTGTTTGGGTTTGGGTTTG	GGCTTGCCTTACCCTTACCCTTACC

	GGTTTGGGTTTGGGTT	CTTACCCTTACCCT
36B4	ACTGGTCTAGGACCCGAGAAG	TCAATGGTGCCTCTGGAGATT
